# Collaboration between employers and occupational health service providers: a systematic review of key characteristics

**DOI:** 10.1186/s12889-016-3924-x

**Published:** 2017-01-05

**Authors:** Jaana I. Halonen, Salla Atkins, Hanna Hakulinen, Sanna Pesonen, Jukka Uitti

**Affiliations:** 1 Finnish Institute of Occupational Health, P.O. Box 310, 70101 Kuopio, Finland; 2 School of Health Sciences, University of Tampere, 33014 Finland, Finland; 3 Finnish Institute of Occupational Health, Uimalankatu 1, 33101 Tampere, Finland

**Keywords:** Collaboration, Employer, Employee, Occupational health service, Workplace

## Abstract

**Background:**

Employees are major contributors to economic development, and occupational health services (OHS) can have an important role in supporting their health. Key to this is collaboration between employers and OHS. We reviewed the evidence regarding the characteristics of good collaboration between employers and OHS providers that is essential to construct more effective collaboration and services.

**Methods:**

A systematic review of the factors of good collaboration between employers and OHS providers was conducted. We searched five databases between January 2000 and March 2016 and back referenced included articles. Two reviewers evaluated 639 titles, 63 abstracts and 20 full articles, and agreed that six articles, all on qualitative studies, met the predetermined relevance and publication criteria and were included. Data were extracted by one reviewer and checked by a second reviewer and analysed using thematic analysis.

**Results:**

Three themes and nine subthemes related to good collaboration were identified. The first theme included time, space and contract requirements for effective collaboration with three subthemes (i.e., key characteristics): *flexible OHS/flexible contracts* including tailor-made services accounting for the needs of the employer*, geographical proximity* of the stakeholders allowing easy access to services, and *long-term contract*s as collaboration develops over time. The second theme was related to characteristics of the dialogue in effective collaboration that consisted of *shared goals, reciprocity, frequent contact* and *trust*. According to the third theme the definition of roles of the stakeholders was important; OHS providers should have *competence and knowledge* about the workplace, become strategic partners with the employers as well as provide quality services.

**Conclusion:**

Although literature regarding collaboration between the employers and OHS providers was limited, we identified several key factors that contribute to effective collaboration. This information is useful in developing indicators of effective collaboration that will enable organisation of more effective OHS practices.

**Electronic supplementary material:**

The online version of this article (doi:10.1186/s12889-016-3924-x) contains supplementary material, which is available to authorized users.

## Background

According to the World Health Organization’s (WHO) Health assembly [1], employees represent half the world’s population and are the major contributors to global economic development. Access to health services is a factor determining employees’ health, in addition to workplace hazards and social and individual factors [1]. Occupational health services (OHS) have a key role in supporting the health and work ability of employees in many settings. Depending on the country and context, OHS may be considered a parallel service provider to the public and private health care sectors or they may have a limited role in supporting employers in ensuring occupational health and safety, or anything in between these. For example, in some countries such as Finland [2] and the Netherlands [3] employers are obligated by law to organize preventive occupational health services for their employees. In addition to preventive services, many employers may also offer medical services (more than 80% in Finland [4]). Workplace health promotion programs have been seen to result in medical cost savings and reduced sickness absences, and are thus beneficial for an organisation’s productivity and economic and staff health outcomes [5].

The changing nature of work means that the role that OHS take and their functions in a workplace also change. Already, the practices at workplaces (e.g. at large and small workplaces) and at different service providers may differ considerably [6]. Regardless of the role taken by OHS, collaboration between OHS and employers is a prerequisite for effective OHS and healthy employees [6, 7]. In addition, good practices as well as collaboration between OHS and employers is essential for effective OHS activity [8, 9], regardless of workplace size. Some information is available about the benefits of good collaboration between the employers, OHS providers, and other stakeholders of occupational health care. These benefits include reduction in sickness absences [8], the possibility to use multiple resources in solving problems, expansion of good practices, and increased trust between stakeholders [9].

We sought to review the available research evidence related to the characteristics of good or functional collaboration in the context of OHS. This summary of information will be useful in the development of indicators of good collaboration that can further help make the arrangement and practices of OHS more effective.

## Methods

### Inclusion criteria

Our research question was: “What are the characteristics of good collaboration between employers and occupational health service providers?” We included studies that examined collaboration between the stakeholders involved in the arrangement of occupational health services. All evidence regardless of the basis on which the OHS was organised (i.e., based on legislations or voluntary) and regardless of the contract type (preventive or curative services) was included.

We included studies reporting qualitative or quantitative results that were published in English between January 2000 and March 2016. We limited the time span to 2000–2016 in order to focus on collaboration within OHS as it is now. While we agree that the earlier debate and conversations about OHS collaboration in literature could be interesting, we felt that in the last 16 years the situation has been fairly stable with the type of collaboration conducted. We considered studies that focused on collaboration itself, not collaboration with respect to particular health conditions. Ethics approval was not needed for a systematic literature review.

### Search strategy

We searched for relevant studies from scientific databases MEDLINE, Scopus, Psych INFO, Social Sciences, and Cumulative Index to Nursing and Allied Health Database (CINAHL), in this order. The search included three terms: (“occupational health” [All fields] AND “collaborat*” [All fields] OR (“cooperat*” [All fields] OR “co-operat*” [All fields]) AND “workplace” [All fields]). We used three separate terms in databases that disallowed using the asterisk (*), for example, for “collaborat*” we used: “collaborate” OR “collaboration” OR “collaborating”. All journal articles and conference proceedings/contributions were included. We complemented the search by articles retrieved by backward referencing and hand searching. Any additional relevant studies identified through hand search or through contacts with experts within the authors’ network were added to the list.

### Study selection

Two researchers (JIH, SA) evaluated all non-overlapping titles from the database searches independently. Based on this initial screening, abstracts for further evaluation were chosen by discussion. JIH retrieved full text articles for the included abstracts, which were again screened independently. Articles chosen for full review were backward referenced for possible additional works to be included.

### Data collection process

After the final selection of articles, JIH extracted relevant information about study context, methods and participants. Data extracts were collected into a separate file and illustrative quotations of the key factors were copied from the articles.

### Quality assessment

The quality of individual studies was assessed using a checklist (Table 1) developed in an earlier review of qualitative studies [10]. The checklist was based on common elements from existing criteria for quality assessment of qualitative studies [11–13]. Two researchers assessed the quality of each included study using the checklist and resolved differences by discussion. No studies were excluded on the basis of quality. Previously, others have found that poorer-quality studies tend to contribute less to the synthesis of results [14], and in the synthesis the findings from the better-quality studies become more important. None of the studies met all quality criteria. Most studies had clear definition of the research questions and aims of the study as well as an appropriate approach to study the research questions. However, most studies lacked clear justification for the use of qualitative approach and the methods used were often inadequately presented.

**Table 1:** Quality assessment of included studies

Quality criterion	Assessment of the six articles		
	Met criterion	Did not meet criterion	Unclear/Cannot say
Is this study a qualitative study?	4		2
Are the research questions/aims clearly stated?	5	1	
Is the qualitative approach clearly justified?	1	5	
Is the approach appropriate for the research question/aims?	5		1
Is the study context clearly described?	4		2
Is the sampling method clearly described?	3	3	
Is the sampling strategy appropriate for the research question/aims?	2	1	3
Is the method of data collection clearly described?	3	3	
Is the data collection method appropriate for the research question?	3		3
Is the method of analysis clearly described?	2	4	
Is the analysis appropriate for the research question?	2		4
Are the claims made supported by sufficient evidence?	4	1	1

### Synthesis of results

As an analytical strategy we used a thematic analysis [15]. This included the identification of major and recurrent themes in the included articles and development of key factors of good collaboration using a ‘constant comparison’ method [16], i.e., by comparing the themes presented in one article with those in others. Comparison of these factors across papers and an attempt to “match” the factors ensured that similar key factors from different papers were captured. For example, in one study theme *long-term collaboration* was mentioned as: “Long-term contracts facilitate continuity and familiarity in relationships” [8] and in another study the same theme was expressed as: “Commitment to a long-term collaboration between the company and the OHS provider” [17]. The selection of the main themes was inductive, starting with data and organizing them into different themes based on terms used in the articles.

## Results

### Study selection

**Fig. 1:**
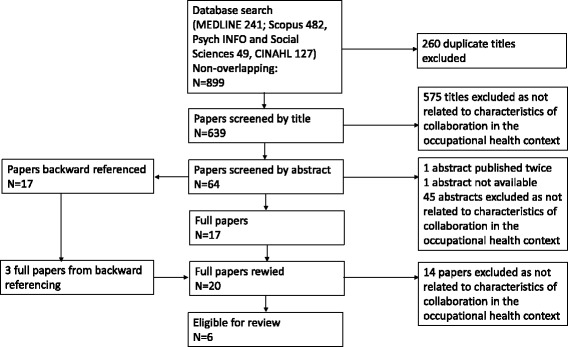
Selection of the reviewed articles

The five database searches resulted in a total of 639 non-overlapping titles (Fig. 1). Of these, 64 were selected based on titles for further evaluation of the abstracts (one abstract could not be found, two titles had the same abstract), and 17 articles were finally chosen for which we obtained full text copies and that were backward referenced [see Additional file 1]. From backward referencing and hand search we identified an additional three full articles that were further included [see Additional file 1], resulting in 20 full articles. After reading these full texts, six articles were considered eligible for the review.

### Study characteristics

The included six articles are introduced in Table 2. Four studies were fully qualitative and were based on face-to-face, telephone, or computer assisted interviews. The numbers of the interviewees varied between the studies from less than 20 to more than 2400. Two studies used mixed methods and their qualitative part was included in the review. None of the included studies were purely quantitative. The number of quality criteria met in each article is also presented in Table 2.

**Table 2:** Characteristics of articles included in the review

Reference	Method	Aim	Quality score ^a^
1. The influence of social capital on employers’ use of occupational health services: a qualitative study. Ståhl et al. 2015 [8]	Interviews: 16 individual interviews, 8 focus group interviews of public sector employees with 44 interviewees, 25 interviews of OHS professionals	To explore how employers and OHS providers describe their business relations and the use of OHS in rehabilitation in relation to the organization of such services.	11
2. Successful collaboration between occupational health service providers and client companies: Key factors. Schmidt et al. 2015 [17]	Semi-structured interviews in 15 companies and their OHS, total of 66 interviews	This paper identifies key factors for successful collaboration between Swedish OHS providers and their client companies	8
3. How can occupational health services in Sweden contribute to work ability? Schmidt et al. 2012 [20]	Semi-structured interviews for 15 companies and their OHS, total of 66 interviews	To identify successful interactions of companies and OHS	7
4. Challenges of OHS for changing working life. Husman and Husman 2006 [18]	Telephone interviews for managers, employees and OHS personnel (*n* = 2438) in 2001 working in a random sample of workplaces	To reveal the expectations of clients of OHS and the factors that are perceived to affect fluency of cooperation.	2
5. Networking between occupational health services, client enterprises and other experts: difficulties, supporting factors and benefits. Peltomäki and Husman 2002 [9]	Mixed methods: computer-assisted telephone interviews (*n* = 6), network interviews (*n* = 6), thematic in-depth interviews (*n* = 6), documents (e.g. annual reports, budgets)	To study difficulties, supporting factors and benefits of networking to enterprises and partners (OHS)	3
6. Towards an effective co-operation between companies and occupational safety and health services. van der Drift 2002 [19]	Interviews in 5 organisations, 4 account managers of occupational safety and health services (OSH-Services), representatives of 3 employer organisations and 1 employee organisation.	From the company point of view, to find out how companies and OSH-Services can co-operate more effectively to obtain a better OSH management system.	3

^a^ Number of quality criteria met in each study, out of 12

### Synthesis of results

We identified three main themes related to the characteristics of good collaboration between OHS and employers (Table 3). These related to 1) time, space and contract characteristics as prerequisite for effective collaboration; 2) characteristics of the dialogue in effective collaboration; and 3) clear definition of roles.

**Table 3:** Factors related to functional collaboration between stakeholders involved in organization of OHS

Theme	Subtheme	Examples of codes included	Further issues related to the factor/theme
Time, space and contract requirements for effective collaboration	flexible OHS/contract	specified set of services; dialogue focused more on the needs at the workplace than on what was included or not in the contract; employers appreciate easy access and flexible ways for contacting OHS; problems can be corrected immediately; flexibility, accessibility and activity of OHS; planning of OHS activities for the workplaces’ needs; tailor-made and flexible services	services developed in dialogue with the employer [8]; more complex relation with frequent contact [20]; regular follow-up and evaluation of companies’ satisfaction with the OHS allows problems to be fixed immediately [17]
	geographical/physical proximity	geographic proximity a central factor for developing a close cooperation; physical closeness	geographic proximity is a central factor for developing close cooperation [8]; employers appreciated easy access and flexible ways for contacting OHS [8]; ease of getting in touch [18]
	long-term collaboration	long-term contracts	provision of OH services is facilitated if the provider has good knowledge of the work conditions and environment in the workplace [8]; collaboration between the companies and the OHS providers develops over time [17]
Characteristics of the dialogue in effective collaboration	shared goals/vision of the future	a shared vision of the cooperation between employers and OH service providers; shared goals, norms and values	the organization and OHS formulate together an agreement on the contribution of the services [19]; selection of suitable partners in relation to goals is important [9]
	reciprocity/dialogue	reciprocity; extensive dialogue; joint commitment; good interaction; two-way communication; discussions and stepwise decision-making	two-way communication in co-operation improves networking [9]; confidence and trust built up in the collaborative process and dialogue [17]
	frequent contact	frequent contact at different organizational levels; continuous dialogue and contact between the company and OHS; active communication; discussions and stepwise decision-making	more complex relation with frequent contact [20]; committed and active focus person [9]; opportunities to follow-up [17]
	trust	mutual trust; important to feel confidence and trust for OHS personnel and their activity; trust and good personal ‘chemistry’; familiarity, build trust	OHS providers contributing to the company’s internal discussions and documents on the work environment as a basis for collaboration founded on trust and confidence [17]; trust helps the functioning of the network, it saves time and increases convenience in cooperation, but is often adhered to individuals [9]
Clear definition of roles	OHS as an expert: OHS has competence, knowledge and skills matching the company’s needs	OHS providers’ insight into conditions in the workplace; good knowledge and understanding of the client; high professional competence of OHS; OHS providers have good knowledge concerning the client workplace; experience, knowledge on and experience with relevant health and safety risks	For the OHS provider it is important to understand the company’s economy and business [17]; OHS needs to transform to meet the demands of more strategic qualities, such as skills in economic, consultative work methods, work organization and profitability [20]; important to ensure high quality training for all OHS experts [18]
	Clear roles preventing overlap and rivalry	HR departments could be considered as rivals when HR services overlapped with the work of OH professionals; clarity about roles were considered important; important to specify and define the role of OHS; organisation formulates its ambitions for OSH-policy including all relevant functions: top management, other managers, internal OSH-staff, employees council	OHS should focus on medical issues [8]; OHS providers need to take a more consultative role in the relationship with their client companies, acting as coaches and assisting the companies to become aware of their own needs and issues [17]; OHS services to work more with prevention as strategic partners, and focusing on “treating the organization”, not the individual [17]

Time, space and contract characteristics: This theme encompassed issues relating to the *flexibility and proximity of OHS to the employer*, as well as the *length of collaboration*. Flexibility was reported as important for effective collaboration by employers, particularly when concerning the functions and being in contact with the OHS. In one article, an illustrative quote from an OHS representative was reported:

“*The collaboration is built on very informal contacts. It is very flexible, and the very best is if the contract with the client company allows an initial free consultation with the OHS provider. In that way, the OHS provider gets information and learns about the company*.” p. 234 [17].

Flexibility also referred to OHS not needing stick to specified contract activities, but addressed employers’ needs of services as the needs emerged. Tailoring services for employers seemed to be a key issue here. Important in flexibility was also feedback and follow-up. Timely follow-up allowed OHS to amend their activities to ensure the client company’s needs being met. Proximity seemed a necessary ingredient for flexibility: having OHS close by was seen as a central factor for developing close cooperation. Physical proximity also enabled flexibility, for example through easy access to the services [8, 18]. The length of collaboration was also important, as that allowed for closer communication and enabled the OHS to be familiar with the employer, their issues, and thus design a tailored approach to each employer.

Characteristics of the dialogue in effective collaboration: Under this theme *shared goals, reciprocity, frequent contact* and *trust* were the key characteristics. To meet the strategic qualities of OHS the stakeholders must have shared goals and vision of the future*.* The goals can, for example, be formulated into a written agreement between the employer and the OHS provider. Joint commitment to collaboration and reciprocity were seen as factors leading to good interaction and further, close and effective collaboration. A quote relating to the importance of frequent contact came from a human resource (HR) manager in one of the articles:

“*The most important thing is, irrespective of the OHS provider, that they try to be close to their clients and to continuously have opportunities to follow-up.*” p. 234 [17].

Dialogue and frequent contact were also seen important for building confidence and trust between the employers and OHS providers. Trust, on the other hand, can increase the convenience in collaboration and improve the use of occupational health services, as an employer had put it:

“*We could be much faster in referring sick-listed employees to occupational health services. It’s all about managers knowing how to consult them and when. We could be much better at using their services. […] It’s about information, but also about relationships, that you have to gain that trust. Our provider is well on the way at getting in much earlier*. (Employer, HR)” p. 7 [8].

When there is enough trust the OHS providers may contribute to the companies’ internal discussions and formation of documents on the work environments [8, 17], which consequently, may help the OHS providers to understand the employers’ goals and the organisation and its functions.

Clear definition of roles: OHS providers were seen as experts that are expected to have *competence, experience and knowledge* on the relevant health and safety issues within the client organisation [8, 18, 19]. Expertise also related to the ability to provide high quality of OHS [9, 18] and to understanding economic factors from the employer’s point of view. High quality training of OHS personnel is important as more and more “OHS needs to transform to meet the demands of more strategic qualities, such as skills in economic, consultative work methods, work organization and profitability” [20]. Having *clearly defined roles* between the stakeholders may prevent overlap and rivalry and thus contribute to smooth and effective collaboration. One quote indicated this clearly:

“*They [the OH provider] get better and better. As an employer, we don’t know so much about medicine, so they do their work on that and do not interfere with employment issues, which they used to do a lot more. Nowadays, their role is to support us in work adjustments and what we need to do. Roles are much clearer now*. (Employer, HR)”p. 7 [8].

One suggestion was offered for how the roles of the employer and service provider can be formulated using a five-step approach. These steps included 1) formation of organisation’s ambitions for OSH policy, 2) formulation of organisation’s expectations concerning the involvement of OSH services, 3) a joint formulation of an agreement on the contribution of the OSH services, 4) recording the agreement in the Service Level Agreement (SLA), and 5) working according to the SLA that is evaluated periodically and adjusted as needed [19]. It was also mentioned that the roles and visions of the employers and OHS providers can be affected particularly by poor collaboration when OHS providers can be seen as self-seekers selling their services, rather than strategic partners helping the organization. With close collaboration the role of the OHS providers can turn into a strategic partner of employers that provide advice and consultation and help employers to see what their real needs are.

## Discussion

### Summary of evidence

Our search indicated that factors related to functional collaboration between employers and OHS providers have been rarely scientifically studied during the past 16 years. In the six studies included in this review three main themes and nine different characteristics were identified as factors of effective collaboration. The first theme consisted of time, space and contract requirements for effective collaboration with three key characteristics: *flexible OHS/flexible contracts*, *geographical proximity* of the stakeholders and *long-term* contracts and collaboration. The second theme was related to characteristics of the dialogue in effective collaboration with *shared goals, reciprocity, frequent contact* and *trust* as the key characteristics. Under the third theme, clearly defining the roles of the stakeholders was emphasized. OHS providers were characterised as *experts* who can deliver high quality services that do not overlap with services offered by the employers, and the role of the OHS providers was expected to become more *strategic* shifting from being curative to preventive service provider.

### Comparison to prior literature

Many of the identified characteristics of effective and good collaboration between stakeholders of OHS agree with those suggested for models of collaboration in other contexts. For example, *shared goals, client-centred orientation, trust, mutual acquaintanceship* (i.e., frequent opportunities to meet) and *information exchange* were presented as indicators of collaboration in the Four-Dimensional Model of Collaboration by D’Amour et al. [21], and *shared goals* and *willingness to cooperate* were included in the Resource Dependence Institutional Cooperation (RDIC) Model by de Rijk and colleagues [22]. Shared goals should be the starting point for collaboration [21], however, this may not always be the case. For example, in a cooperative project aimed at returning employees to work after sickness absence, the participants were found to have two different views on the goals of cooperation; some of the participants saw collaboration as a new approach to rehabilitation, whereas others saw it as a way of rendering the existing organisations more efficient [23]. Although forming and understanding the goals may take time [24], they are likely to improve the results of collaboration.

Frequent contacts and information exchange may result in positive experiences of collaboration, which may in turn improve the perception of the other collaborators and thus increase one’s willingness to collaborate [22] as well as team spirit [24]. In an experiment of team communication, proactive communication regarding information about the next goal was a factor increasing efficiency of team performance [25]. People are also likely to get to know each other better through frequent contact, which is a prerequisite for *trust*, another characteristic of effective collaboration mentioned not only in the articles included in this review but also other articles with different contexts of collaboration [21, 26, 27]. Gaining trust along with competence and knowledge of OHS are likely to derive from long-term collaboration that we observed as another prerequisite of effective collaboration. Although long-term collaboration was seen beneficial, it can be difficult to attain continuity if the contracts have to be frequently re-negotiated [8].

According to a recent study from Belgium trust and reciprocal knowledge (i.e., better understanding of the roles and tasks) between general practitioners, occupational health physicians, and social insurance physicians is important for effective sick leave management [28]. However, the authors also called for changes in political and economic structures, including common training involving interdisciplinary collaboration, to improve collaboration between physicians. Also elsewhere in the health care sector, clear roles have been found essential for effective multidisciplinary collaboration [29], which agrees with the current findings.

Regardless of the several commonalities with collaboration models in general, collaboration between employers and OHS providers has some specific characteristics. On one hand, employers are expected to identify OHS collaboration as an important part of their strategy along with occupational safety issues. OHS providers, on the other hand, are expected to primarily serve the client company, but also individual employees, and thus need to have contact with both stakeholders.

### Practical implications

Some guidelines for establishing collaboration between employers and OHS providers could be developed based on the previously suggested models of collaboration and our current findings. For the employers, it would be beneficial to first formulate 1) their OHS policy and 2) expectations regarding occupational health services. In the first contact with employers, the OHS provider could undertake the following steps: 1) agreeing the frequency and content of discussions with the employer and 2) setting up structures that make conversation with the OHS convenient. Together these stakeholders should agree on the goals of collaboration and determine the roles of each operator. Thereafter, regular liaison and follow-up between the stakeholders will increase knowledge and understanding of each other as well as trust.

### Strengths and limitations

The main strength of this study is, that to our knowledge, this is among the first studies summarizing evidence regarding collaboration between employers and OHS providers. We have summarized findings mainly from qualitative studies, however, evaluation whether evidence from each of these studies is “good” is difficult. Often, the proposed sets of standards do not distinguish between issues relating to the quality of reporting and those relating to design and execution [15]. A recent study suggested 12 main criteria for assessing qualitative studies, but concluded that the finer definitions of these criteria, or the importance of them, are not easily agreed on, especially between health science fields [30]. However, we made an effort to assess the quality of each included study using a checklist developed for the assessment of qualitative studies [10]. Some of the obvious gaps in reporting include lack of detailed information about the justification for the use of qualitative methods as well as lack of description of the method of analysis or sampling methods and data collection. For example, often it was not clear what was asked in the interviews or whether the interviews were conducted similarly for all participants of one study. In addition, two articles may have reported on the same data [17, 20]. It is clear from this study and our cursory quality assessment that there is both a need for further quantitative studies on collaboration between OHS and other stakeholders as well as a need for further, good quality, qualitative studies. However, we are likely to have missed some important studies where collaboration has not been the outcome but a key factor to another outcome. These may include studies on the effectiveness of worksite interventions [31, 32]. Also the selection of the search terms is likely to have affected the selection of the studies. For example, using synonyms of workplace, like worksite, could have resulted in slightly different list of studies. During preparation of the search strategy we experimented with a number of different synonyms. Based on these experiments we suspect that we have found most of the relevant studies. The employer and OHS should have the same goals, and benefits are often tripartite. Therefore it is necessary to have successful collaboration in order to achieve the common goals. As a consequence, it is obvious that most interventional studies of occupational health provide practises of good collaboration between participating partners but collaboration has rarely been mentioned as key word, and quality of collaboration has not been described. In addition, our selected databases could have been added to by others. In order to counter this effect, we performed a cursory search in the Social Science Citation Index. This search was sensitive, but not specific, and our additional screening revealed no additional articles. Further, all of the identified articles included in this study were European, and mainly from the Nordic countries, suggesting a lack of OHS or OHS collaboration evaluation from outside Europe. Because of the wide variation in how occupational health care is organised in different countries, more studies on collaboration between OHS and stakeholders are needed from outside Europe to form a broader picture of the phenomenon. OHS collaboration with employers in Finland, for example, is very extensive - OHS conduct workplace investigations that include familiarization of the occupational nurse and occupational physician to the conditions at workplace. Thus, in addition to physical risk assessment, OHS can suggest actions that aim to improve workers’ health physically and psychosocially. Another example is statutory collaboration between the employee, employer and OHS when an employee’s sickness absence has prolonged. All three stakeholders are required to meet to discuss possibilities of work modifications to help the employee to return to work. These extensive collaborations may be rare outside the country [33], but other types of collaborations may exist that should be evaluated and reported.

## Conclusions

This systematic review on the key characteristics of effective collaboration between the employers and OHS providers included six articles. These articles provided evidence for several factors that have been regarded beneficial for collaboration between the employers and OHS providers. Many of the factors were common with those previously suggested in the more general models of collaboration, thus, interventions that improve the effectiveness of collaboration in other contexts may be transferable to OHS. Although the existing literature regarding OHS is limited, the information provided in this article can be used in the development of indicators of good collaboration that can help make the arrangement and practices of OHS more effective.

## Additional file

Additional file 1: List of 17 articles chosen for backward referencing and full evaluation based on abstracts. (DOCX 21 kb)

## Abbreviations

OHS: Occupational health service
OSH: Occupational safety and health
