# Supplementary material for: Collaboration between employers and occupational health service providers: a systematic review of key characteristics
Source: BMC Public Health. 2017 Jan 5;17:22. doi: 10.1186/s12889-016-3924-x (PMC5217434; doi:10.1186/s12889-016-3924-x)
Supplement: Additional file 1: — List of 17 articles chosen for backward referencing and full evaluation based on abstracts. (DOCX 21 kb) [file 12889_2016_3924_MOESM1_ESM.docx]

**Additional file 1**

Collaboration between occupational health service stakeholders: a systematic review of key characteristics

Jaana I. Halonen, Salla Atkins, Hanna Hakulinen, Sanna Pesonen, Jukka Uitti

List of 17 articles chosen for backward referencing and full evaluation based on abstracts. Those whose title is bolded (3) were included to the review and the rest were excluded.

1. Kraaijeveld RA, Schaafsma FG, Ketelaar SM, Boot CR, Bültmann U, Anema JR. Implementation of the participatory approach for supervisors to prevent sick leave: a process evaluation. Int Arch Occup Environ Health. 2016 Mar 12 [Epub ahead of print].
2. Ståhl C, Åborg C, Toomingas A, Parmsund M, Kjellberg K. **The influence of social capital on employers' use of occupational health services: a qualitative study**. BMC Public Health. 2015;15:1083
3. Schmidt L, Sjöström J, Antonsson AB. **Successful collaboration between occupational health service providers and client companies: Key factors**. Work. 2015;51(2):229-37.
4. Hannon PA, Hammerback K, Garson G, Harris JR, Sopher CJ. Stakeholder perspectives on workplace health promotion: a qualitative study of midsized employers in low-wage industries. Am J Health Promot. 2012;27(2):103-10.
5. Auvinen AM, Kohtamäki K, Ilvesmäki Msc A. Workplace health promotion and stakeholder positions: a Finnish case study. Arch Environ Occup Health. 2012;67(3):177-84.
6. Seing I, Ståhl C, Nordenfelt L, Bülow P, Ekberg K. Policy and practice of work ability: a negotiation of responsibility in organizing return to work. J Occup Rehabil. 2012;22(4):553-64.
7. Lindberg P, Vingård E. Indicators of healthy work environments--a systematic review. Work. 2012;41 Suppl 1:3032-8.
8. Schmidt L, Sjöström J, Antonsson AB. **How can occupational health services in Sweden contribute to work ability?** Work. 2012;41 Suppl 1:2998-3001.
9. Lin YK, Lee LH, Sheu SH. An integrated occupational health consultation model for the medical supply manufacturing industry. Ind Health. 2010;48(6):749-57.
10. Naumanen P, Liesivuori J. Workplace health promotion activities of Finnish occupational health nurses. Public Health Nurs. 2009;26(3):218-28.
11. Wachs JE. Building the occupational health team: keys to successful interdisciplinary collaboration. AAOHN J. 2005;53(4):166-71.
12. Eriksson, A., Axelsson, S.B., Axelsson, R. Collaboration in workplace health promotion - A case study. International Journal of Workplace Health Management, 2012;5(3):181-193.
13. Broberg, O., Hermund, I. The OHS consultant as a facilitator of learning in workplace design processes: Four explorative case studies of current practice. International Journal of Industrial Ergonomics, 2007;37(9-10):810-816.
14. Blizzard, K. The meaning of occupational health among human resources professionals--a qualitative study. AAOHN journal: official journal of the American Association of Occupational Health Nurses., 2006;54(6):282-289.
15. Husman, P. Occupational health services in Finland-do they work well? Opinions of the managers. International Congress Series, 2006;1294:45 -48.
16. Peurala, M., Kankaanpää, E. Developing occupational health services for social and health care employees in municipal organisations. International Congress Series, 2006;1294:73-76.
17. Nishikido, N., Yuasa, A., Motoki, C., Tanaka, M., Arai, S., Matsuda, K., Ikeda, T., Iijima, M., Hirata, M., Hojoh, M., Tsutaki, M., Ito, A., Maeda, K., Miyoshi, Y., Mitsuhashi, H., Fukuda, E., Kawakami, Y. Development of multi-dimensional action checklist for promoting new approaches in participatory occupational safety and health in small and medium-sized enterprises. Industrial Health, 2006;44(1):35-41.

Articles found through backward referencing the 17 articles. Those three whose title is bolded were included in the review and the fourth was excluded.

1. van der Drift DW. **Towards an effective co-operation between companies and occupational safety and health services**. Int J Occup Med Environ Health. 2002;15(2):179-83.
2. Husman K, Husman P. **Challenges of OHS for changing working life**. International Congress Series. 2006;1294[0]:19-22.
3. Peltomäki P, Husman K. **Networking between occupational health services, client enterprises and other experts: difficulties, supporting factors and benefits**. Int J Occup Med Environ Health. 2002;15:139–145.
4. Linnan L, Bowling M, Childress J, et al. Results of the 2004 National Worksite Health Promotion Survey. Am J Public Health. 2008; 98:1503–1509.
